# Product News

**DOI:** 10.1155/S1463924678000188

**Published:** 1978


### Product News

*Editor's Note:—*
*The address of the manufacturer appears in italic at the end of each item. In some cases this address will be that of a subsidiary to the manufacturing company as the address given is that from which the information has been obtained.*

### Easy-to-use scientific computer

Digital Equipment Corporation have recently introduced the latest in their range of scientific computers. Aimed basically at the first time buyer with little or no experience of computers or electronics, it offers a cheap simple but effective entry into on-line computer data processing. The computer system known as MINC, is based on the PDP 11/03 processor and comprises a choice of seven additional function modules. The software is an easy to learn extension of PDP-11 basis. It permits the user to issue commands to mass storage devices and to control sampling and output through analogue and digital interface. The use can very simply collect data and apply various data processing criteria to it, develop histogram, perform fast fourier transforms and the like. Compatability with existing DEC labs computers as the broad range of PDP-11 processors is retained by the ability to run RT-11 FORTRAN.

The versatile system is equally applicable to the laboratory or desk situation. It cost approximately £8.5k for a basic configuration including a 32k word PDP-11/03 with extended and floating point instruction sets.

Three serial line inputs and an IEEE 488 standard bus interface enable as many as 14 laboratory instruments and testing devices to be handled on-line. A double-density dual diskette unit for program and data storage and a new video graphic terminal, the VT105 are included. The terminal has full alphanumeric capabilities plus a graphics facility of 512 horizontal by 250 vertical positions.

Any combination of seven functional modes, up to a maximum of eight, can be added in order to arrive at a customised laboratory system. The module available are analogue-to-digital converter, digital-to-analogue converter, digital input, digital output, multiplexer, preamplifier, and programmable clock. The input-output modules allow multiple channels, ranging from four to 64, depending on module type and use. To meet different application requirements the modules have been designed for easy reconfiguration by the user. Connection of module to instrument is by a simple screw terminal plug which eliminates rewiring and gives the MINC system maximum mobility. A series of schematic cards allow the user to check a configuration prior to connecting physically to the computer. Any combination that is now allowable is simply indicated at this stage.

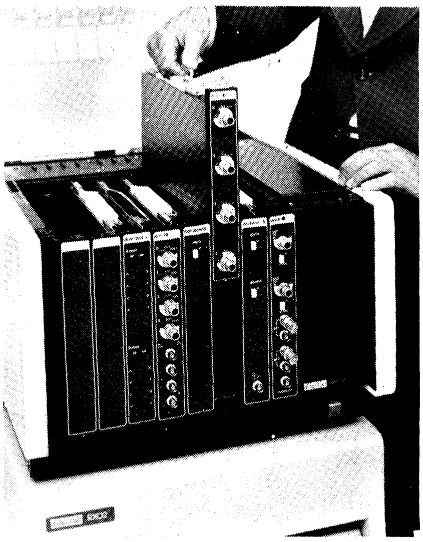

*MINC, the new mobile, easy-to-use laboratory system from Digital Equipment Company.*

*Digital Equipment Co. Limited, Digital House, Kings Road, Reading, Berks, UK.*

### Chemical processor and analyzer

A new microprocessor-based sample processor and analyzer, suitable for a wide range of chemical analysis and chemical or biological processing procedures, has been introduced by Instrumentation Specialities Company. Marketed as the ChemResearch Model 1560, it is capable of automating complex processes in research and quality control laboratories.

A simple, versatile, sample-handling module is combined with a sophisticated controller utilizing an integral microprocessor-based computer. The user can easily develop his own processing or analyzing programs and quickly enter them with simple keystrokes on the controller's keyboard. A panel displays the keystroke options available for each program step entry; programming is no more difficult than, for example, a calculator. Looping and subroutine capabilities minimize program steps for repetitive procedures.

The sample changer can handle test tubes or tissue culture plates. Up to 210 samples can each be processed through 960 discrete steps involving programmed events — for example, reagent addition from more than 40 sources, agitation, incubation, control of reaction times, and withdrawal of samples for analysis.

Operation can be with or without the sample changer, allowing use of the controller alone for single-batch processes and for closed-loop systems. The processor can be interfaced with other instruments to measure pH, optical absorbance, or other characteristics, and can automatically vary the program according to measured results.

A built-in interface for a teletype and computer/desk calculator allows additional data input and output, and enables the operator to analyze data during program execution. Programs can also be stored on magnetic tape.

A brochure giving full details is available.

*Instrumentation Specialities Company, P.O. Box 5347, Lincoln, Nebraska 68595, USA.*

### Midget air valve

A manual/mechanical microvalve capable of at least ten million operations on completely oil-free air has been introduced by Joucomatic Controls Ltd. Intended for pneumatic automation and control applications, the Series 310 is an NC 3/2 model (normally-closed, 3 port 2 position) with a choice of operating head. Ports are tapped G⅛ (the modern designation for ⅛ inch BSP) and they can be fitted with quick-connect push-in couplings for 4 mm outside diameter plastics tubing. The valve is dimensionally interchangeable with those of other manufacturers.

A zinc-alloy diecasting forms the valve body, and the midget spool is in acetal resin with nitrile p-rings. A special feature is that the exhaust is fully closed before the supply begins to open, thus conserving air when the valve is used as a limit switch with a slow rising ramp.

Operating heads include a palm button and a toggle switch for manual use, and roller levers of both plastics and metal for mechanical actuation.

The 310 valve is also available in universal form (either normally-closed or normally-open simply by changing tube connections). These have light-alloy bodies.

*Joucomatic Controls Ltd, Air Automation House, Navigation St, Wolverhampton, UK.*

### Single-board computer

A new single-board computer, the iSBC 80/30, from Intel has a number of new features which are said to provide the user with a great deal of flexibility in the way a system is configured. The computer is designed to be used in a Multibus system where it can be one of up to fifteen other processors on the same bus. In such a system each processor has its own individual memory and I/0 and can share a common (system) memory and I/0 structure. The 16K byte read/write memory on the iSBC 80/30 board is system memory, local memory or both. This is achieved by providing the memory with two access ports.

The iSBC 80/30 is the first Multibus compatible computer in the iSBC range to use Intel's 8085A microprocessor. It is also the first to employ a slave microprocessor (an Intel 8041 or 8471 UPI) for I/0 processing.

Software support for the iSBC 80/30 includes a new version of the powerful RMX-80 multi-tasking executive PL/M-80, Ans Fortran 77 and the 8085 micro assembler.

*Intel Corporation (UK) Ltd., 4 Between Towns Road, Cowley, Oxford, OX4 3NB, UK.*

### Microprocessor control and sequencing system

GEC-Elliott Process Automation Ltd have introduced a control and sequencing system package, Marcus 16, for applications which are more complex than can be catered for by the programmable logic controller (PLC) and yet, costwise, do not justify the use of a mini computer system. A single Marcus 16 is suited for applications requiring the control of eight to twelve loops and a number of associated sequencing actions. It can be used for continuous control, logic and sequencing, alarm monitoring (both analog and digital), data acquisition and recording either on an individual stand-alone basis or as a hierarchical system.

The system is designed to be extensible so that a basic package can itself be enlarged or additional Marcus 16 packages can be added to a plant at any time to form a hierarchical system.

*GEC-Elliott Process Automation Limited, New Parks, Leicester, LE3 1UF, UK.*

### Automatic titrator

EDT's new ATC 700 automatic titration system has been developed to remove the drudgery from titrimetric analyses. Titration is still a widely used analytical procedure in many areas of industry and research because the inherent accuracy of such an analysis is high and the method quite simple. However, it is a time-consuming procedure and a considerable degree of expertise is required by the analyst to obtain the 0.5 - 1% precision of which the method is capable. The ATC 700 contains a microprocessor (INTEL 8080) which takes over the time consuming part of the titration and presents the results directly in volume units to the analyst at the end of the titration. The procedure is as follows:—

—The operator loads a sample and inserts electrodes
—The operator presses one button
—The instrument carries out the complete titration, locates the end-point, displays the answer and refills the burette ready for the next analysis.
—The operator writes down the answer and loads next sample.

The word 'operator' has been used deliberately instead of 'analyst' since the simplicity of the instrument allows less skilled workers to achieve the same high precision as a practised analyst.

The ATC 700 can perform a wide variety of titrations including acid/base, weak acid/strong base and vice versa, and chloride/silver titrations.

*EDT Research, 14 Trading Estate Road, London NW10 7LU, UK.*

### Microprocessor-based digital thermometers

Fluke International Corp. have announced the introduction of a series of microprocessor-based digital thermometers. Two models are currently available. These are the 2180A, which is designed for applications using platinum or nickel resistance sensors, and the 2190A which is intended for use with thermo-couples. Both offer the facility of switch selectable °C or °F temperature registration. The use of a microprocessor in their design provides significantly greater application flexibility, and enables the highest accuracy levels to be achieved.

Model 2180A offers switch selectable temperature resolution of 0.01° 0.1° for temperatures below 204°C, and auto-ranging to 0.1° from 204°C to full scale. It can handle the complete negative and positive temperature ranges measurable by 100-ohm platinum resistance, and 100-ohm or 120-ohm nickel resistance temperature sensors. Additionally, the 2080A has a 0 to 1000-ohm resistance range for the testing and calibration of RTDs. A multipoint selector option consisting of two internally located switches, enables the user to read up to five of each of two types of RTD, or ten of one type.

Model 2190A is available in two versions, each of which offers a resolution of 0.1° over the complete thermocouple temperature range. One version is designed for use with thermocouple types J, K, R, T, or C, and the other with thermocouple types J, K, R, S or E. The specific thermocouple type is manually selected by means of a switch on the instrument's plug-in input module. In common with the model 2180A, a multipoint selector option is available for the 2190A, for the selection of thermocouple types. The 2190A may also be used with a combination battery pack and stable d.c. supply voltage from -10mV to +100mV, to simulate any thermocouple input. This enables the instrument to compare, calibrate or check any other thermocouple whatever its location.

Options common to both instruments include a high/low limits comparator, which incorporates a digital thumbwheel switch for selection of mode, polarity, normal and limit temperatures, and provides a visual indication of temperature abnormalities; analogue/digital auxiliary output, for use with external equipment such as a printer or chart recorder; an alarms output module containing four independent comparators for signalling up to four stages of high and/or low out-of-limit conditions; and a rechargeable battery pack.

Included in Fluke's series of microprocessor-based digital thermometers is a module designed for the calibration of both analogue and digital thermometers. The Model Y2003 calibrator module is used in conjunction with Fluke's 2190A digital thermometer to form an integrate calibration system. The calibrator module generates an extremely stable, high resolution d.c. voltage, which is supplied in parallel to both the 2190A and the instrument to be tested or calibrated. This voltage may then be adjusted to simulate temperatures over the total input range of the unit under test, thus providing an accurate and measurable comparison check with the 2190A. Also, a fixed offset value can be selected to check the response of the U.U.T., and linearity at any specific point.

*Fluke International Corporation, Garnett Close, Watford WD2 4TT, UK.*

### X-ray energy spectrometer

The Kevex-Analyst 6600 is a micro-computer based x-ray energy spectrometer that quantitatively analyses atomic elements in bulk specimens. A video monitor instantaneously displays alpha-numeric symbols listing the elements present together with associated weight per cent concentrations. Hard copy of each analysis is supplied via a built-in line printer. Each analysis is nondestructive, rapid (on the order of 20 seconds) and requires little or no sample preparation. A combination of excitation methods consisting of three radioisotopes plus a low powered 30 kV x-ray tube is employed to fluoresce specimens being analysed. NIM standard modules provide flexibility and adaptability to a wide range of dedicated analytical tasks.

*Kevex Corporation, 1101 Chess Drive, P.O. Box 4050, Foster City, California 94404, USA.*

### Organics-in-water analyses

It is safe to say that difficulties in complying with requirements of the US Clean Water Act have resulted, in part, from the scarcity of instruments capable of performing the required analyses and the cost of such analyses. Finnigan

Instruments have taken a step toward eliminating this bottleneck with the announcement of their new automated OWA-Series GC/MS systems.

The OWA-Series features pre-programmed parameters built into the data system specifically to handle all organics-in-water problems. This simplifies operation (modest training requirements make a specialist unnecessary) and it speeds analysis throughput to accommodate the large number of samples required. Equally important, the initial cost of the system is significantly lower than that of previous instruments capable of meeting EPA requirements.

*Finnigan Instruments, 845 West Maud Avenue, Sunnyvale, California 94086, USA.*

### Microprocessor-controlled spectrometer for cost effective x-ray analysis

The recently introduced Philips PW 1400 sequential X-ray fluorescence spectrometry system, which operates under total microprocessor control, combines the flexibility of a research instrument with the ability to function fully automatically for routine industrial analysis.

The design differs markedly from previous spectrometers has improved analytical performance is easier to use and has increased measurement speed.

Its modular construction enables the basic spectrometer to be adapted to meet specific customer needs.

The well proven goniometer, sample chamber and optics of the successful Philips PW 1410/PW 1450 range have been retained, the latest electronic developments are employed to give faster measuring times, with reduced interference and improved background correction.

A universal interface is provided to permit connection to a range of computer facilities such as a programmable calculator, a mini-computer or on-line to a central computer. Full software support packages have been developed for use with Philips, DEC mini-computers, and a Hewlett Packard calculator.

The PW 1400 is controlled by a microprocessor and manual controls are virtually eliminated. Operation of the spectrometer is conducted through a question-and-answer dialogue using a teletype printer or VDU unit. With simple mnemonic commands, built-in verification procedures and a clear synoptic display on the front panel of the spectrometer, the system facilitates error-free operation by even relatively unskilled staff.

Optional automatic sample handling accessories include a six-position sample loader and a fully automatic sample changing system.

The pneumatically operated changer is based on a system completely new to X-ray spectrometry. This is the "programmable tray" principle, initially developed by Philips to enable large batch handling on nuclear analysis instruments.

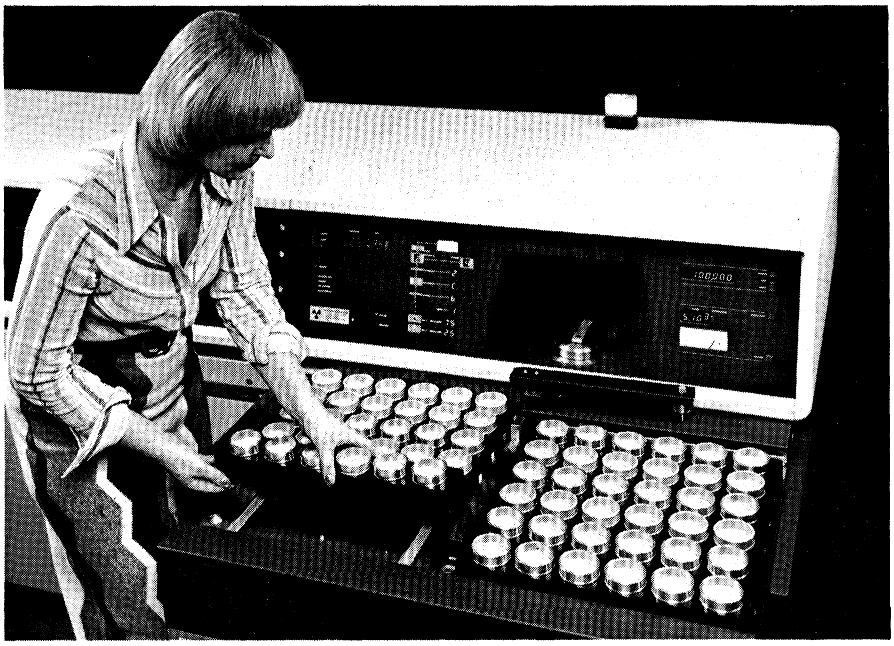

*Operator removing a sample tray from the automatic sample changer of the Philips PW1400 sequential XRF spectrometer.*

Up to 72 samples at a time can be placed in the sample changer, using a series of six-sample trays. Simple, programmable identification cards slotted into each tray give positive identification of the sample and define the required analysis programme. Coupling to a continuous conveyor belt feed is also practical.

The Philips PW1400 also allows simultaneous XRF and XRD measurements.

*N. V. Philips' Gloeilampenfabrieken, Science & Industry, TQ III-3, Eindhoven, The Netherlands.*

### Computer-controlled liquid scintillation counter

Packard Instrument Ltd. has introduced a new computer-controlled liquid scintillation counter where the only user interaction is loading and unloading trays of samples. Automatic efficiency correlation (AEC) ensures that all samples are measured with maximum figure of merit, plus optimum radionuclide separation in dual-label counting. The versatility of this programmed instrument and its operational simplicity are key features of this answer system. The 15 different measuring programs are stored on a flexible diskette. Program selectors are clipped on the side of the servo-tray by the user to ensure that correct user parameters are selected from the diskette c¾ storage to measure the batch of samples. There are 9 trays, each can be measured, interrupting a run of samples, with automatic return to the original counting sequence.

Defining or modifying a program and listing all programs is a simple question and answer routine conducted through the conversation print terminal. Samples activity is computed from efficiency correlation data automatically established by the system. Correlation curves are plotted, using a quench indicating parameter or external standard channels ratio, or a number derived from the measurement of an external standard source.

System calibration control (SCC) is provided to maintain the integrity of the efficiency correlation and the AEC. An active parameter control allows modification to the parameters of a sample being measured to determine the best channel settings. Data reduction includes percentage of standard and peak integration.

*Packard Instrument Ltd., Caversham Bridge House, 13-17 Church Road, Caversham, Berks., RG4 7AA, UK.*

### Enzyme analysers

The new microprocessor based Eppendorf photometer type PCP 6121 is fully programmable and can store up to 12 routine kinetic enzyme methods all of which can be recalled or changed by pressing two buttons on the simple keyboard.

Enzyme activity is measured by pressing the "start" button and thereafter the photometer constantly monitors and checks linearity for the duration of the programmed measuring interval.

An acoustic signal indicates the end of the measurement period and the activity in u/l appears automatically on the large digital display. An error control lamp lights up if any of three control measurements deviate more than ±10% from the calculated total activity. These control activities can of course be recalled in order that a decision can be made. In addition to measuring enzyme activity the PCP 6121 can store and run up to 12 end point methods all of which can be initiated at the touch of a button. Concentration for end point methods are determined by means of the stored computation factor or the standard concentration which is averaged from the three standard measurements. The error control lamp lights up if any of the standard concentrations deviate more than ±5% from the average value. By pushing a button you can still recall the standard photometric absorbances, declare as valid the measured values and then begin the measuring series.
*Anderman & Company Ltd., Central Avenue, East Molesey, Surrey KT8 0QZ, UK.*

### Remote computer access

Data Dynamics have introduced a new concept in remotely accessing a computer. Known as Tele-ZIP, the new equipment is easily portable and allows the user to communicate with a computer from any location where there is a telephone line and a standard television receiver or video monitor.

Tele-ZIP is housed in a suitcase and comprises a typewriter-style and an acoustic modem. In use, Tele-ZIP is connected to any standard television receiver's aeriel socket using the cable supplied or, if required, can be connected to any video monitor requiring a video signal at IV peak-to-peak. The computer is dialled on the telephone and, once the carrier is received, the telephone hand-set is placed in the acoustic modem's receptacle within Tele-ZIP. The Tele-ZIP is now on-line and forms a complete video display terminal.
*Data Dynamics Limited, Data House, Springfield Road, Hayes, Middlesex, UK.*

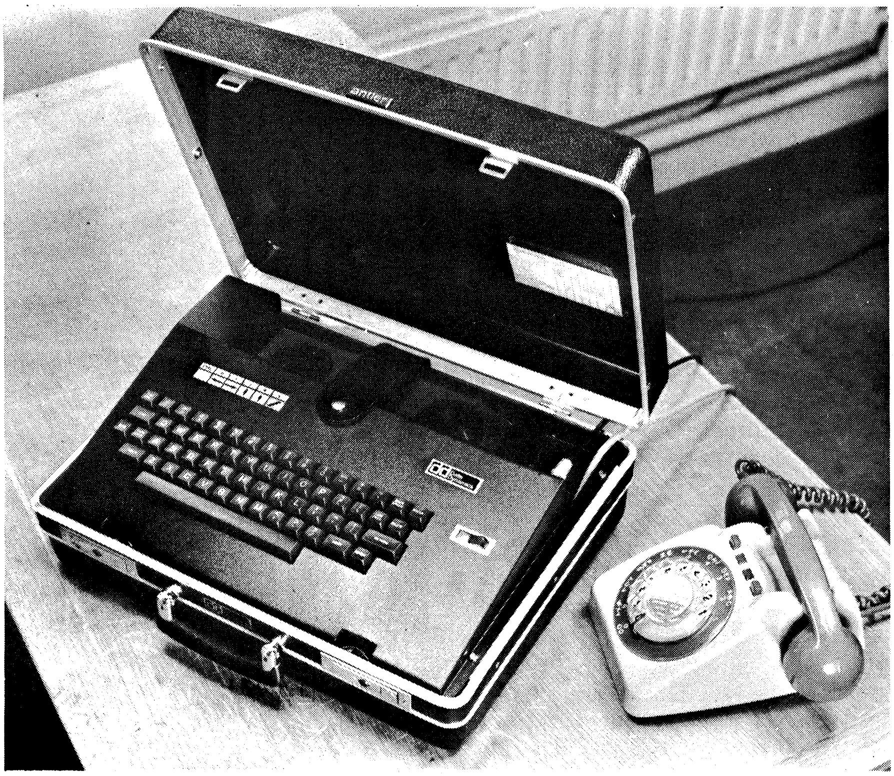

*The Tele-ZIP.*

### EPE-dedicated data acquisition system

Varian has introduced the E-900, said to be the first EPR data acquisition system offering microprocessor technology in a versatile data management system. The E-900's simple controls give the operator easy access to a read/write memory of 23K bytes and a magnetic tape storage of 250K bytes with program editing, programming, plotting and matrix manipulation.

Using simple commands on the E-900's keyboard, the operator can easily: accumulate a single- or multiscan time-averaged EPR spectrum; store spectra complete with all parameter information on tape; print out file header information of selected spectra; display up to three spectra and perform X-Y scale shift, linear baseline correction, differentiation, integration, noise spike deletion, g-value calculation, and many other manipulations; "zoom in" on any region of a spectrum; perform Stone-Maki simulation of multiple spin systems and display results.

With little prior programming experience, operators can write their own programs with the E-900. Further, one may accumulate either single- or multi-scan spectra in memory; store spectral information on magnetic tape complete with parameter details; locate a spectrum on tape files and plot it, print out header information, and display both spectrum and header on the CRT. As many as three spectra, together with descriptive labels, can be displayed for comparison.
*Varian AG, Steinhauserstrasse 8, CH-6300 Zug, Switzerland.*

### Automatic continuous colorimetric analyser

The Burkard Automatic Continuous colorimetric analysis system — BACCA — offers microanalysis by automated continuous colorimetric chemical analysis. All manual analytical methods normally used for water, boiled water, sea water and waste water are available. BACCA is a mobile laboratory centred around the Model CF-2 colorimeter designed in conjunction with British Gas Corporation Ltd. Up to 6 channel per unit are available to provide continuous monitoring to user specification. It is capable of working up to 400 metres from the sampling site and for up to 28 days continuously before reagent refill is required. Its versatility is highlighted by the range of proven methods available. These include silica, hydrazine, phosphate, chloride, nitrite, total phenol, sulphate and total alkalinity measurements. In most cases BS 2690 is adapted to fit in with established analytical techniques.

The signal outputs of each channel on the colorimeter represents the ionic concentration, these are scanned and printed automatically at ½-hour intervals and identified with the date and time of analysis. Parallel BCD outputs are available to communicate with external data processing facilities.
*Burkard Ltd., P.O. Box 25, Rickmansworth, Herts., UK.*

### GC/MS system

New features have been added to the Hewlett-Packard 5992 benchtop gas chromatograph/mass spectrometer (GC/MS) to improve the instrument's performance, increase its reliability and provide unattended operation once a sample is injected. The HP 5992B, a more powerful version of the earlier desktop-computer controlled GC/MS, offers automatic valves, higher ion source temperature and expanded software.

The compact GC/MS system, which features automatic tuning, an efficient hyperbolic quadruple mass filter, electron multiplier detector and a micro-processor-controlled GC, is designed to provide quantitative and qualitative identification of unknown chemical compounds. The HP 5992 system consists of two modules; one contains the thermal printer/plotter, the other houses the GC and mass spectrometer subsystems. Unattended operation is made possible with valves that can be opened and closed automatically under software control during both the autotune calibration and data acquisition phases.

This feature reduces errors and frees the operator for other tasks. The valves protect the system since they automatically close in case of power failure. Assuring correct valve timing prevents pressure surges that could damage electronic components or contaminate the analyzer. New and improved software also has extended the capabilities of the HP 5992B which can detect GC peaks and automatically correct for background interference. Less time is required to identify components since the instrument can perform on-line library searches during data acquisition and print the names of the identified compounds on top of the GC peaks. The desktop computer can scan on-line standard libraries containing up to 50 common pollution and drug spectra that are stored on cartridges. For applications where expanded spectra storage and increased speed of data retrieval are desired, optional flexible disc drives are available.

Standard data acquisition and display software provide total ion chromatograms and enable the user to scan and store individual mass spectra which can be plotted and tabulated off-line when needed. Selected ion software allows the system to monitor and record up to six different ions simultaneously.

*Hewlett-Packard Limited, King Street Lane, Winnersh, Wokingham, Berkshire, RG11 5AR, UK.*

### Automatic purge and trap sampler for GC and GC/MS

An automatic purge and trap sampler which concentrates traces of volatile organic compounds to levels detectable by gas chromatographs (GC) and gas chromatograph/mass spectrometers (GC/MS) has been introduced by Hewlett-Packard. It is especially useful in the detection of minimal levels of volatile organic compounds in municipal drinking water as recommended by many government authorities. It is also suitable for the analysis of beverages, urine, slurry materials and many other aqeous samples.

The HP-7675A purge and trap sampler uses the dynamic headspace technique in which the sample is continuously purged by gas flowing through it. Volatile organics in the sample are transferred to the gaseous phase and adsorbed on a trap. The trap is then rapidly heated to desorb the volatile organics and they are flushed into the analytical column, which may be a packed or capillary column. While the chromatographic analysis proceeds the trap is heated to a high temperature and backflushed to remove any residues before the next adsorption cycle.

The unit is programmable, allowing the user to select purge time, trap desorbtion time and temperature as required for the analysis. For ease of operation, infrequently used controls are recessed behind the sampler's front panel. After the sample has been loaded the front panel start run button is pushed to initiate unattended operation. A complete analytical cycle generally takes less than 35 minutes.

*Hewlett-Packard Limited, King Street Lane, Winnersh, Wokingham, Berkshire, RG11 5AR, UK.*

### U-chamber chromatograph for simultaneous multiphase circular chromatography

A new version of the CAMAG-U-Chamber system based on the design of R. E. Kaiser for fast optimization of HPTLC is available. It has four solvent capillaries, each fed by its own dosage syringe in addition to the central solvent capillary of a regular U-chamber. This multiphase U-chamber quickly optimizes the solvent development by running a circular chromatogram with 4 different solvents simultaneously. The chromatographic effect of all mixtures between two neighbouring phases can also be seen. The instrument also serves as a regular 10 cm U-chamber.

The 5-solvent system U-chamber unit is operated by the same control unit that is part of a regular U-chamber chromatograph. The central solvent capillary can be fitted with a sample injection valve.

*CAMAG AG, Homburgerstrasse 24, 4132 Muttenz, Switzerland.*

### Remote spectrophotometer

Applied Colour Systems have recently introduced a remote spectrophotometer system, which is a stand-alone software and hardware package for high speed, high resolution, remote colour measurement in the paint, textile, ink and plastic industries. The sytem can be used either in the 'stand-alone' mode without interfacing to a main computer, or combined with existing ACS-500 or ACS-600 systems to give complete remote spectro-operation with identical performance to the basic system.

The ACS remote system contains an ACS Spectro-Sensor, a DEC PDP 11/03 computer processor and a DEC LA-36 DEC writer II. In addition to data reduction, tristimulus computations, instrument calibration and colour difference calculations. The system, which through the Chroma-Pac software, provides a complete colour control capability can also be operated remotely.

The ACS Spectro-Sensor has scanning speeds of 2 to 6 seconds (4 seconds average) and monitoring the lamp intensity allows direct feedback control. It measures reflectance and transmittance to better than +0.2 FMCII/colour difference units. Accuracy is achieved by taking 1000 discrete measurements across the visible spectrum, and converting the data to either 16 or 31 point wavelength information through the 400 - 700nm spectrum.

*Applied Colour Systems Inc., Princeton New Jersey, USA.*
*UK agent: BOC Limited, Hammersmith House, London W6 9DX.*

### Data acquisition system

DIAC (Distributed Intelligent Acquisition and Control) is a new high speed system from Newport Instruments, which uses a single coaxial cable 'ringmain', linked to central microprocessor-based master station, to monitor and control process plant functions over a radius of 5KM. The very latest LSI and CMOS circuitry is used and transmission on this data highway is extremely fast — 25,000 bits/second. The system is modular with a potential of up to 10,000 channels so up to 4000 analogue or 65,000 digital input/output channels (or any equivalent combination) can be provided.

The unique use of a single coaxial cable as the data transmission highway, which can send information bi-directionally, shows considerable savings compared with normal plant cabling, and also ensures reliable data flow in noisy industrial environments. Interconnection of all units by a serial 'daisy-chain' principle greatly simplifies control room equipment design and installation and allows pre-wired modular plant to be simple plugged into the coaxial cable. The 'DIAC' system, originally designed by Carlo Gavazzi, can also be economically front-end a process computer.

*Newport Instruments Limited, Newport Pagnell, Bucks. MK16 9HF, UK.*

### Automatic serum dispenser

An automatic serum dispenser unit, the Seromat is now available from Dynatech Laboratories, which offers a considerable saving of time, together with versatility and consistent accuracy. Up to sixty 0.002ml volumes of typing sera can be dispensed into the corresponding wells of a pre-oiled Teresaki tissue-typing plate. Push-button simplicity of operation means that the complete cycle time for loading a plate, dispensing the serum into the wells and unloading the plate is less than 5 seconds.

The Seromat dispensing head consists of 60 micropipettes grouped to match the format of a Teresaki plate. Each micropipette has its own 200 micro-litre reservoir and, once the pipettes are primed with a panel of typing sera, up to 200 plates can be seeded in under 30 minutes. Accuracy is equal to conventional manual filling, but speed is immensely greater.

A great advantage of the system is consistency and versatility. Each successive sample is placed in exactly the same place in each well and each plate is identical to the one before. At the same time, the plate can be filled with any predetermined pattern of sera. Volumes are dispensed with consistent accuracy. A further advantage in serum economy is provided by the fact that after a batch of tissue-typing plates has been seeded, unused serum can be collected easily from the micropipette reservoirs and returned to the master reservoir for storage.

Also available from Dynatech is a compact oil dispenser which operates on a similar principle to the Seromat to pre-oil the Teresaki plates. Pre-oiling is essential if evaporation of the sera is to be prevented. The oil dispenser can oil 400-500 plates per hour with precise, predetermined oil levels. Operation is quick and simple and variations in dispensing volumes can be adjusted to suit particular test requirements.

*Dynatech Laboratories Ltd., Daux Road, Billingshurst, Sussex RH14 9SJ. UK.*

### Micro-electronic, digitally controlled syringe injection pump

A micro-electronic, digitally controlled syringe injection pump, designated the Model 1001 Micro-jector, has been introduced by Houston Atlas, Inc. The Micro-jector accurately delivers precise quantities of fluids or gases in discrete steps at a selected constant rate from a hypodermic syringe. Among the many uses for the Micro-jector, are: injection of liquid or gas samples into analytical devices; a precise means for controlling linear motion; in the fast mode, it can be used for rapid injection in chromatography.

All movement is regulated from a lighted push-button console, where a single knob controls the rate of motion. The direct-drive system is simple and reliable, with just two moving parts — the rotor and the carriage. A non-rotating drive screw advances the carriage. A non-rotating drive screw advances the carriage 1/768th of an inch per pulse, at precisely timed rates ranging from 10-pulses per second to 1-pulse every 27 seconds. Rate of volume delivered is determined by the syringe size. For example, a 100 *ul* syringe will deliver average volumes as low as 0.13 *ul* per minute, up to 35.0 *ul* per minute, and be completely adjustable over this entire range.

The uncomplicated self-adjusting syringe holder readily accepts all syringe sizes up to and including 50 ml. Carriage stops are automatic; after the front limit stop has been set and the first shot has

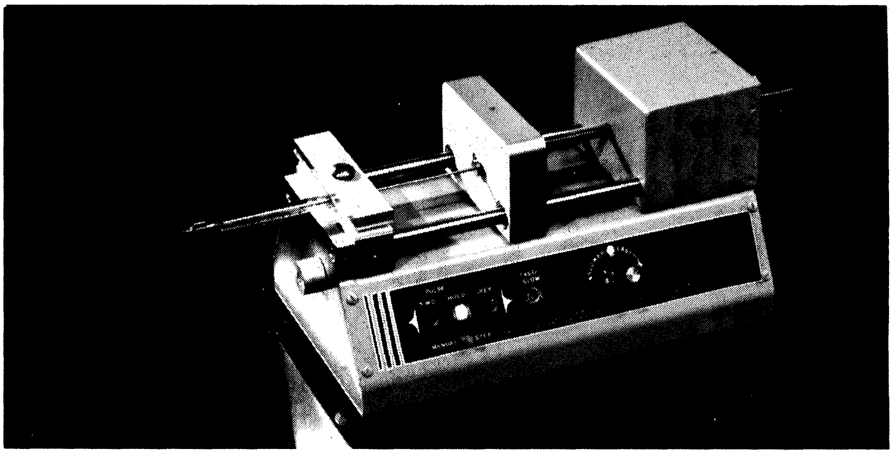

*Model 1001 Micro-jector syringe pump*

been completed, the carriage automatically retracts at high speed to permit instant, easy accessibility to the syringe. The manual controls on the lighted console compliment this feature for greater versatility and facile operation.

Problems of backlash, binding, and accidental bumping of the syringe plunger are eliminated as a result of the Micro-jector's rigid construction, smooth carriage operation with precision positioning, and the strong stepper-motor with up to 23 lb of thrust.

*Houston Atlas, Inc., 9441 Baythorne Drive, Houston, Texas 77041, USA.*

### Dissolved oxygen electrode

The latest electrode in the Orion range of chemical sensing electrodes is the model 9708 dissolved oxygent electrode. This electrode greatly simplifies the measurement of dissolved oxygen, especially biological oxygen demand measurement of dissolved (BOD). The Orion electrode works on a similar principle to the familiar Clark electrode, but goes one step further. The current produced in proportion to the oxygen concentration is converted to potential by means of a small electronics package built into the electrode. As BOD measurements fall within the range of 0 to 14ppm the pH scale of a pH meter can conveniently be used; the electrode can therefore be used with any pH meter.

*Orion Research Inc., 380 Putnam Ave., Cambridge, Ma 02139, USA.*
*UK agent: MSE Scientific Instruments, Manor Royal, Crawley, West Sussex, RH10 2QQ.*

### GC with built-in data reduction

Packard's range of gas chromatographs has now been expanded to include the 430 series GC with built-in dual integrator and printing facilities.

The new system is based on the microprocessor controlled 429 GC, which Packard launched at the 1977 "Het Instrument" exhibition in Amsterdam. In the 429 series the microprocessor was mainly used to control and optimize the analytical performance of the system. However, in the 430 dual GC, processor capacity has been expanded to perform independent dual channel integration of the detector signals and to produce a complete analysis report via a built-in printer.

The dual integration function is especially attractive when combined with the Multi Dimensional SwitchingLSystem (MDSS) module. This module contains all required hardware, including the injectors, columns and detectors, to perform column switching techniques such as solvent flushing, backflushing, heart-cutting and selective sample transfer. Each of these functions is pre-programmed and can be incorporated in any GC program the operator wants to carry out. Eighteen complete GC programs of 15 time-based instructions each can be stored and executed at the touch of a button. The column switching technique employs solenoid valves which alter pressures at various points in the chromatographic system to change flow rates and reverse flow directions, thus eliminating leakage and overcoming the temperature limitations of conentional valves.

The system has interchangeable analytical modules. Injection ports, columns and detectors are combined into one integral module, which can easily be exchanged to suit a particular analysis.

The integration and reporting of the 430's two independent channels has been achieved by the addition of only 5 extra keys. Via the keyboard the operator can select the desired program as well as the format of the post-run print-out. A visual display allows checks to be made on both sensed and entered data at all times during the operation.

*Packard-Becker B.V., Vulcanusweg 259/P.O. Box 519, Delft, The Netherlands.*

